# A Randomised Pilot Trial to Demonstrate the Feasibility of a Prototype Electronic Heating Device in Patients with Meibomian Gland Dysfunction

**DOI:** 10.3390/biomedicines13122952

**Published:** 2025-11-30

**Authors:** Jacqueline Tan, Tianni Jia, Sidra Qamar, Jennie Diec, Fiona Stapleton

**Affiliations:** 1 School of Optometry and Vision Science, UNSW Sydney, Sydney, NSW 2052, Australia; tianni.jia@unsw.edu.au (T.J.); sidraqamar26@gmail.com (S.Q.); f.stapleton@unsw.edu.au (F.S.); 2 nthalmic Pty Ltd., Sydney, NSW 2019, Australia; j.diec@nthalmic.com

**Keywords:** comfort, meibomian gland dysfunction, electronic heating, warming, eyelid

## Abstract

**Objectives**: To compare the safety and efficacy of a prototype electronic heating device, Meiboleyes^®^, with the BRUDER Moist Heat Eye Compress for the treatment of Meibomian Gland Dysfunction (MGD). **Methods**: Adults with evidence of active MGD (Ocular Surface Disease Index [OSDI] score ≥ 13, fluorescein tear break-up time [TBUT] < 10 s and meibomian gland secretion score ≤ 12 for 15 glands of the lower lid) were enrolled in this prospective, randomised, parallel group, investigator-masked dispensing study (Australian New Zealand Clinical Trials Registry–ACTRN12624000175572). Meibomian gland secretion (MGS) score and number of meibomian glands yielding liquid secretion (MGYLS), lipid layer thickness, TBUT, ocular physiology and subjective symptoms were measured at baseline, and 2 weeks and 6 weeks following treatment. Linear mixed model analysis was conducted to compare the two groups and changes over time. **Results**: Ten participants (average age 38.7 ± 14.5 years) in the Meiboleyes^®^ test group, and 10 participants (average age 38.9 ± 14.8 years) in the BRUDER control group completed the study. MGS and MGYLS significantly improved in both treatment groups from baseline to the 2-week and 6-week follow-up visits (*p* ≤ 0.006). Significant improvements in TBUT (5.5 ± 1.8 vs. 8.3 ± 2.1 s, *p* = 0.044), OSDI scores (45.2 ± 15.1 vs. 27.4 ± 12.9, *p* = 0.027) and visual analogue scale dryness (55.3 ± 27.2 vs. 28.0 ± 23.9, *p* = 0.023) were observed in the Meiboleyes^®^ group only after 6 weeks of treatment. No other significant differences were observed over time or between groups. Eight treatment-related adverse events were reported in the Meiboleyes^®^ group compared to seven in the BRUDER group. All resolved without sequalae. **Conclusions**: The prototype Meiboleyes^®^ device was safe and effective for use as an at-home treatment for MGD when used twice daily for six weeks. Improvements in meibomian gland function were comparable to the BRUDER Moist Heat Eye Compress, but significant improvements in tear film stability and subjective comfort after 6 weeks of treatment were observed in the Meiboleyes^®^ group only.

## 1. Introduction

Clinically significant meibomian gland dysfunction (MGD) affects up to 25% of the population above 40 globally [1]. MGD is described as “a chronic, diffuse abnormality of the meibomian glands, commonly characterised by terminal duct obstruction and/or qualitative/quantitative changes in the glandular secretion. This may result in alteration of the tear film, symptoms of eye irritation, clinically apparent inflammation, and ocular surface disease” [2]. Older age increases risk of MGD [1,3]. The primary role of the meibomian glands is to secrete lipids and proteins to the ocular surface, to promote tear film stability and reduce evaporation [4]. Meibum proteins also regulate the proliferation of corneal epithelial cells, contributing to ocular surface homeostasis [5].

MGD is typically treated with warm compresses and a wide variety of in-office treatments, including device-driven technologies to warm the eyelids [6]. Lid warming therapy involving the application of heat and massage is the mainstay of clinical treatment for MGD [7] and has been used for the past 40 years [8]. The application of heat melts the solidified oils and the massage helps to remove the stagnant oils from the glands.

Warm compresses using moist towels heated in a microwave are commonly recommended but are a poorly standardised treatment for MGD as patients have no means of discerning the appropriate starting temperature of the warm compress to enable delivery of the therapeutic effects at the level of the glands. This optimum therapeutic temperature is 41.5 °C [9]. For warm compresses using moist towels, this is achievable by heating the towels to 45 °C prior to placing on-eye. This method will allow the inner eyelid temperatures to reach therapeutic levels after approximately 4–6 min and be sustained, provided the towels are replaced with a freshly heated towel every 2 min [10]. However, this method is very laborious and cumbersome for patients to follow and would require a second person to help warm the moist towels for replacement every 2 min. Otherwise, the patient will be required to remove the towel from their eyes, preventing a sustained temperature delivery for the length of the treatment. 

Owing to this cumbersome method, several manufacturers have developed eye masks containing various materials designed to be heated with a microwave (MGDRx EyeBag [11] (The EyeBag Company, Halifax UK), MediBeads [12] (Bruder Healthcare Company, Alpharetta, GA, USA); gel beads–TheraPearl [13,14] (Bausch & Lomb Inc., New York, USA); flax seeds–Blepha EyeBag [15] (Theà Pharma, Clermont-Ferrand, France)) or without (warming wafers–EyeGiene [16] (Eyedetec Medical Inc., Danville, CA, USA); heated disc–TearRestore (https://www.tearrestore.com/thermalmask, accessed on 15 October 2025)). These eye masks have been designed to be heated/activated only once to deliver heat required for one treatment session, removing the cumbersome task of reheating. However, some drawbacks of these convenient methods include, again, the patient not knowing if they have the correct starting temperature. Although instructions are given on the length of heating time in the microwave, due to varying microwave wattage, this may vary the result. A study performed on several different eye masks including the BRUDER, TheraPearl, Blepha EyeBag, EyeGiene and the moist towel method, found that only the cumbersome Bundle method using microwave warmed layers of moist towels kept in a covered dish was able to reach the therapeutic temperatures required [12]. In addition, these tests were performed under ideal conditions with methods for ensuring the correct manufacturer heating guidelines were followed. In a patient’s home, the final eyelid temperatures are likely to be even lower. Another known clinical drawback with the prescribing of lid warming therapy or any treatment is compliance. Eye care practitioners typically prescribe an initial lid warming therapy of 10–15 min, twice per day. Even with the advent of convenient microwaveable eye masks, remembering to conduct the treatment within the patient’s busy lifestyle can be difficult, with practitioners only able to rely on verbal questioning on whether the treatment had been performed.

Although microwaveable eye masks have their drawbacks, they are a cost-effective (approx. AUD 50) and convenient at-home solution for patients with MGD. Alternatively, in-office treatments such as the LipiFlow Thermal Pulsation System [17], Intense Pulsed Light (IPL) therapy alone [18] or combined with radiofrequency [19] or low light therapy [20] or Quantum Molecular Resonance [18,21] are effective and can limit the drawbacks of microwaveable eye masks. Compliance is considerably better given patients are not required to perform the treatment themselves. LipiFlow is typically a one-off treatment [22], while 3 to 4 IPL treatments are common [23], with further treatments with these devices given as required. The major drawback of these in-office treatments is cost—with one treatment of LipiFlow costing around AUD 1500 and a series of IPL costing around AUD 1000. Furthermore, not all clinics offer these services due to high initial equipment outlay and hence accessibility can be problematic.

Meiboleyes^®^ is a prototype electronic eye warming device designed to address the drawbacks of current options. It is an electronic device which requires minimal input from the patient to heat the device to the desired temperature and delivers the heat constantly, to allow for the required therapeutic temperatures to be sustained over the treatment period. It is designed to be used at-home, giving it the convenience of current microwaveable eye masks without their current issues in delivering and sustaining therapeutic temperatures. Being an at-home device, it is more convenient and accessible than Lipiflow and IPL (with or without adjunctive treatments) and the costs are projected to be only slightly higher compared to microwaveable eye masks. 

Therefore, the aim of this project was to evaluate the safety and efficacy of the Meiboleyes^®^ device (test) compared to the BRUDER Moist Heat Eye Compress (control) for the treatment of signs and symptoms of MGD. The BRUDER Moist Heat Eye Compress was chosen as the control for this pilot study as it is a popular microwave heat mask recommended by eye care professionals in Australia, where the study was conducted. In addition, the BRUDER Moist Heat Eye Compress demonstrated the highest heat retention capability when compared with other commercially available eyelid-warming masks in an ex vivo study [24].

## 2. Materials and Methods

### 2.1. Study Design

**Figure 1:**
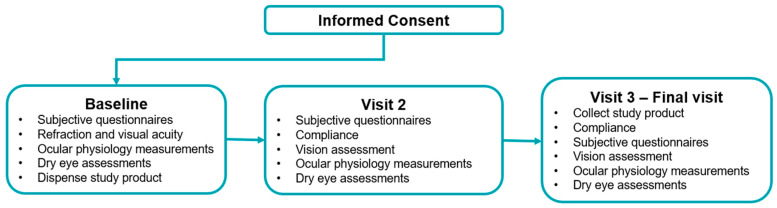
Study design flowchart.

The study (Figure 1) was approved by the University of New South Wales Human Research Ethics Committee (iRECS5657) and conducted in accordance with the Declaration of Helsinki. The trial was registered on the Australian New Zealand Clinical Trials Registry (ACTRN12624000175572) on 23 February 2024. Recruitment was conducted through the School of Optometry and Vision Science, UNSW, using ethics-approved advertisements displayed on university noticeboards and websites. Written informed consent was obtained from all participants prior to commencement of any study-related procedures. Enrolments commenced on 4 June 2024, and the last participant’s last visit was conducted on 7 November 2024.

### 2.2. Study Participants

Sample size calculation was not performed for this pilot study. The sample size (*n* = 10 in each group) was determined empirically. Participants were eligible to participate if they met all of the inclusion criteria and none of the exclusion criteria. Inclusion criteria included adults aged ≥ 18 years; willing to comply with the allocated treatment for the duration of the trial; best-corrected visual acuity of 20/40 or better in each eye; evidence of meibomian gland obstruction (based on a meibomian gland secretion score of ≤12 for 15 glands of the lower lid in both eyes at the baseline visit [17]); Ocular Surface Disease Index [OSDI] questionnaire score ≥ 13 [25] and fluorescein tear break-up time [TBUT] < 10 s in both eyes [26]. Exclusion criteria included any uncontrolled ocular disease (except for MGD and dry eye disease associated with MGD) or uncontrolled systemic disease; corneal abnormality or disorder that impacts normal spreading of the tear film (keratoconus, pterygia, scarring) or corneal integrity; current use of punctal plugs, anticipated insertion during the study, or a history of punctal cautery in either eye at any time or anticipate such a procedure during the study; LipiFlow® or other professional in-office lid-heating therapy, meibomian gland probing, or therapeutic gland expression in either eye within 6 months prior to baseline; MGD treatments including warm compress therapy, eyelid hygiene, eyelid massage, and manual lid expression within 2 weeks prior to baseline; contact lens use anticipated during the study; Meibography—greater than 75% partial glands at the baseline visit [27]; known allergy to spandex or silicone (due to materials used in the devices); pregnancy or breastfeeding. 

### 2.3. Study Treatments 

A description of the study treatments is shown in Table 1.

Participants were asked to use their allocated study product twice per day, for 15 min each time. The allocated treatment was dispensed at baseline according to the predetermined randomisation table (https://www.graphpad.com/quickcalcs/randomize1/, accessed on 15 October 2025) and the same device was used throughout the study period for each participant.

The test product Meiboleyes^®^ (nthalmic Pty Ltd, Sydney, NSW, Australia) was fitted to each participant by the unmasked investigator. The fabric cover came in three sizes (10 mm, 15 mm and 20 mm) and together with an adjustable headband was fitted to accommodate varying anatomical differences, ensuring an optimal customised fit for each individual. The device required daily charging via a USB-C cable provided. Once turned on, the device would warm up and was ready for application after approximately five min. The device was placed over the closed eyes and held in place with the adjustable head band. Built-in audio cues and coloured indicator lights provided updates on battery and charging status, warm-up progress, and treatment completion. The device automatically powered off at the end of the treatment session. The fabric cover and the silicone eye cups could be cleaned by wiping with alcohol swabs or moist tissues if required. 

The control product BRUDER Moist Heat Eye Compress (Bruder Healthcare Company, Alpharetta, GA, USA) is one-size-fits-all and no fitting was required. The mask was microwaved for 20 to 25 s and then placed over the closed eye and held in place with the adjustable headband for 7.5 min. The mask was reheated in the microwave for another 10 s and then placed over the eyes for an additional 7.5 min. The mask could be hand-washed when visibly soiled, dried and re-used.

### 2.4. Study Parameters

The primary objectives were to compare the change in meibomian gland secretions, tear break-up time and ocular physiology (corneal staining, conjunctival redness and keratometry) between the test and control treatments. The secondary objectives were to compare subjective symptoms as measured by The Ocular Surface Disease Index and visual analogue scales (VAS), and lipid layer thickness between test and control. The order of assessments was conducted from the least to the most invasive. 

#### 2.4.1. Questionnaires 

The Ocular Surface Disease Index questionnaire (Allergan Inc, 1995, Irvine, CA, USA) and 100-mm visual analogue scale symptom surveys relating to comfort and vision where a score of 0 represented poor/worst imaginable and 100 being excellent/perfect were self-administered at the start of each visit. 

#### 2.4.2. Tear Film Characteristics 

Tear film lipid layer thickness (LLT) was measured with the LipiView Ocular Surface Interferometer (Johnson & Johnson, Irvine, CA, USA) [28]. The average, minimum and maximum values were recorded, and values of 100+ were considered as 101 nm for the analysis. 

Non-invasive keratography break-up time (NIKBUT) was measured with the Oculus Keratograph 5M (Oculus, Arlington, WA, USA). For each eye, three measurements were taken for each eye, and both the first break and average break were recorded. The repeated measurements for first and average break were averaged for each eye for the analysis.

Fluorescein tear break-up time was evaluated by applying a sodium fluorescein strip (OptiStrips-FL100, Optimed, Lane Cove West, NSW, Australia) wetted with preservative-free unit dose saline (0.9% sodium chloride; Pfizer Inc., Bentley, WA, Australia) with excess fluid shaken off [29]. The tear film was viewed with a yellow Wratten filter (No. 12; Kodak) and cobalt light of the slit-lamp biomicroscope (Zeiss SL-120; Carl Zeiss Meditech, Jena, Germany).

#### 2.4.3. Meibomian Gland Function 

Meibomian gland secretion (MGS) score: 15 meibomian glands of the lower eyelid were expressed using the Meibomian Gland Evaluator (Johnson & Johnson, Santa Ana, CA, USA), which applies a standardised pressure on the eyelid [17]. The quality of secretion expressed was given a meibum score—0 = no secretion; 1 = inspissated consistency; 2 = cloudy liquid secretion; 3 = clear liquid secretion—and the sum of the grades for all 15 glands assessed was the MGS score (range 0–45) [17].

Meibomian gland yielding liquid secretion (MGYLS) score: the number of glands with cloudy or clear liquid secretion (range 0–15) were counted [30]. 

Meibography: images of the oil-producing glands in the upper and lower eyelids were obtained using the Oculus Keratograph 5M (Oculus, Arlington, WA, USA) [31]. Meibomian gland loss was graded using the five-point Meiboscale [27].

#### 2.4.4. Safety Measures 

At each visit, keratometry readings were obtained using the Nidek TONOREF III (Nidek Co., Ltd., Aichi, Japan). Bulbar and limbal conjunctival redness, and overall extent of fluorescein corneal staining were assessed by slit lamp biomicroscopy examination. Clinical features were graded based on the CCLRU grading scale [32] (a 0 to 4 scale where 0 = none, 1 = very slight, 2 = slight, 3 = moderate and 4 = severe, with grades in 0.5 steps used in this study). 

### 2.5. Statistical Analysis

Data analysis was performed using IBM SPSS Statistics (Version 30) (SPSS Inc., Chicago, IL, USA). 

Normality of data was assessed using the Shapiro–Wilk test and mean ± SD were reported. Baseline demographics were compared using either independent *t*-test or Mann–Whitney U test depending on data distribution. Variables that were not normally distributed were log-transformed prior to analysis. A linear mixed model with random effect for individuals was run for each group. Fixed effects of study visit, treatment group and their interaction were included with Bonferroni adjustment for multiple comparisons. Statistical significance was set at *p* < 0.05.

Data from both eyes were collected. No significant differences were found between the two eyes. Therefore, the right eye was selected for reporting other endpoints.

## 3. Results

Of 22 eligible participants, two withdrawals occurred, both from the BRUDER treatment group (one adverse event–severe transient blur following application–and one personal reason). Baseline demographics for the 20 participants who completed the study and were included in the analysis are shown in Table 2.

### 3.1. Meibomian Gland Function

**Figure 2:**
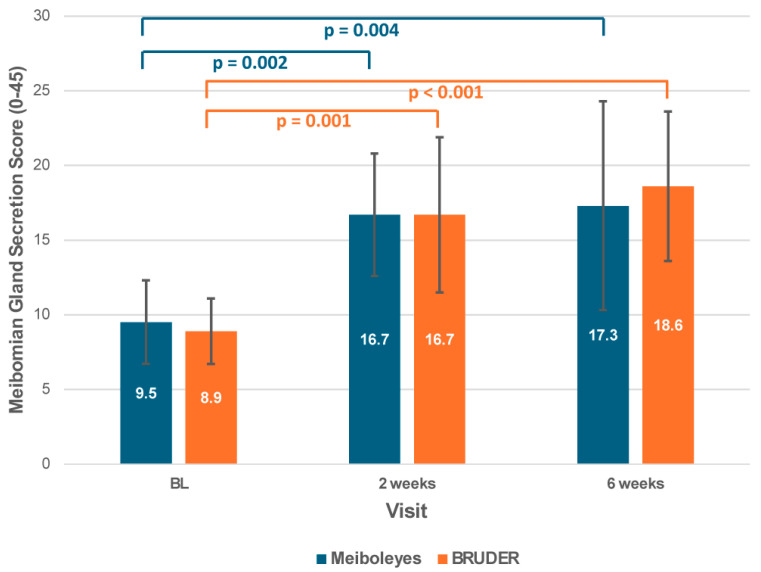
Meibomian gland secretion score for the Meiboleyes^®^ and BRUDER treatment groups at each visit.

Meibomian gland secretion scores significantly improved from baseline to the 2-week and 6-week visits for both treatments (*p* < 0.001; Figure 2). However, there was no significant difference between the two groups (*p* = 0.78).

**Figure 3:**
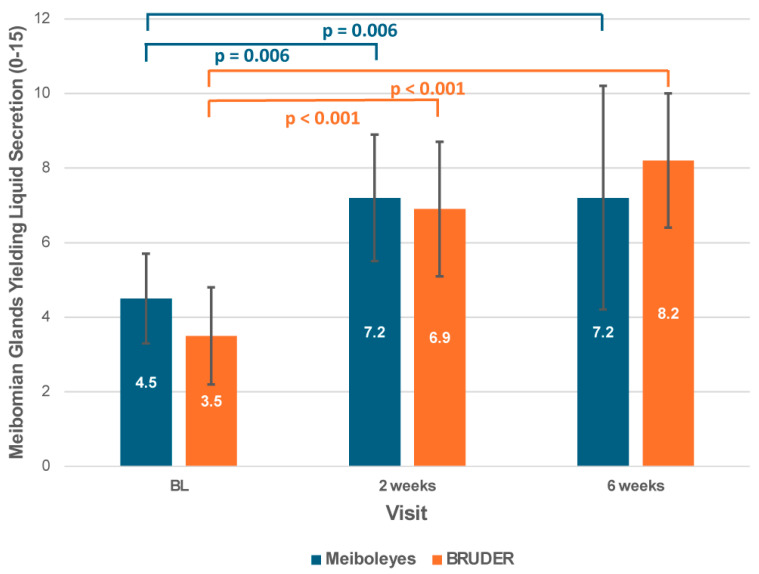
Meibomian gland yielding liquid secretion score for the Meiboleyes^®^ and BRUDER treatment groups at each visit.

Similarly, the number of meibomian glands yielding liquid secretion significantly improved from baseline to the 2-week and 6-week visits for both treatments (*p* < 0.001; Figure 3), but there was no significant difference between the two groups (*p* = 0.84).

There were no significant differences between the two treatment groups in meibography score for the upper or lower eyelids at the baseline and 6-week follow-up visit (*p* ≥ 0.31) or between visits (*p* ≥ 0.87).

### 3.2. Tear Break-Up Time

**Figure 4:**
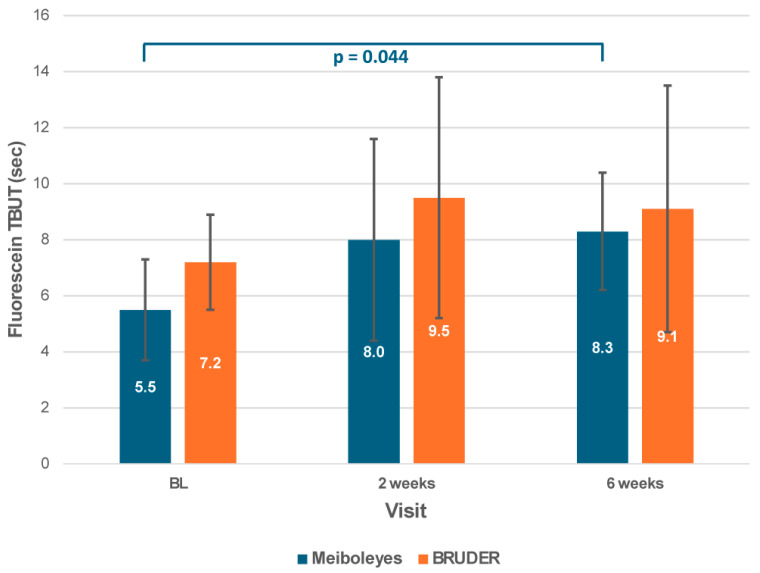
Fluorescein tear break-up time for the Meiboleyes^®^ and BRUDER treatment groups at each visit.

There were no significant differences between the two treatment groups in first or average NIKBUT at any visit (*p* ≥ 0.84) or between visits (*p* ≥ 0.67). However, while there was no significant difference in fluorescein TBUT between the groups (*p* = 0.12), fluorescein TBUT significantly improved between baseline and 6 weeks for the Meiboleyes^®^ group only (post hoc *p* = 0.044; Figure 4).

### 3.3. Ocular Physiology 

No significant changes in keratometry readings were observed over time for either treatment group (*p* ≥ 0.96). Although mild improvements in overall bulbar and limbal conjunctival redness were observed over time (*p* ≤ 0.036), the changes were not statistically significant after adjustment for multiple comparisons (*p* > 0.05). The levels of fluorescein corneal staining in both groups were very low, with no significant differences between groups (*p* = 0.94) or between visits (*p* = 0.75). 

### 3.4. Subjective Symptoms

**Figure 5:**
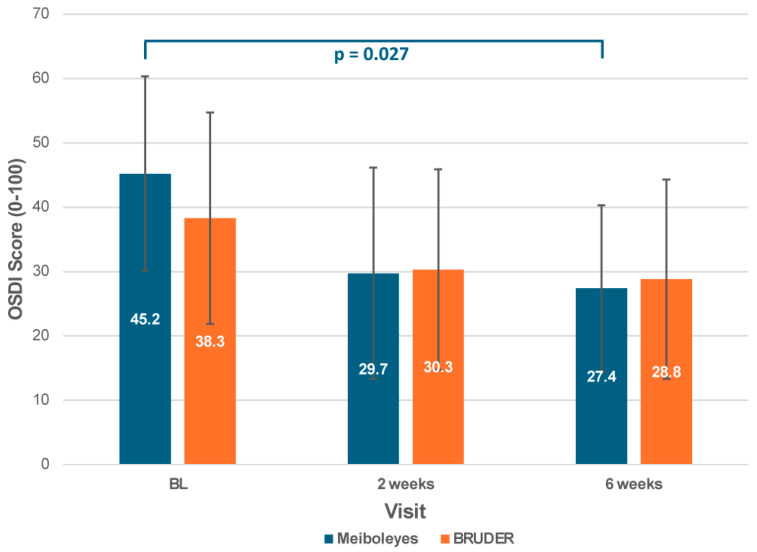
Ocular surface disease index scores for the Meiboleyes^®^ and BRUDER treatment groups at each visit.

**Figure 6:**
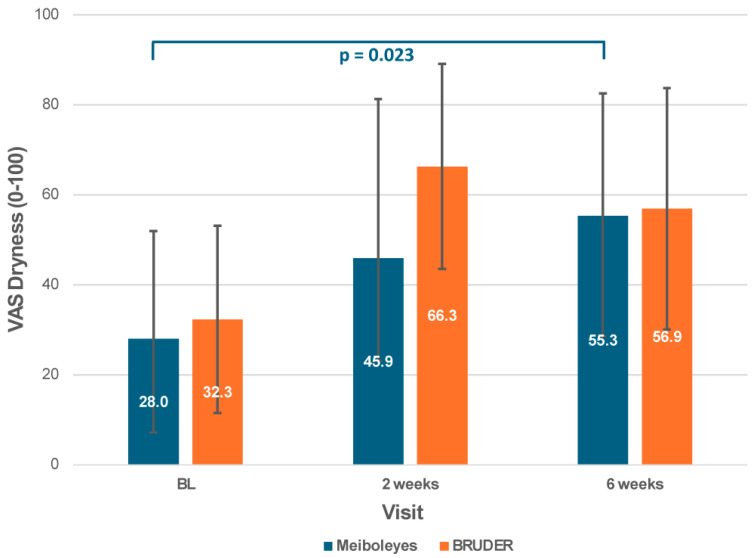
Visual analogue scale—dryness scores for the Meiboleyes^®^ and BRUDER treatment groups at each visit.

Significant improvements in subjective comfort were observed in the Meiboleyes^®^ group only after 6 weeks of treatment compared to baseline, as measured by the OSDI questionnaire (27.4 ± 12.9 vs. 45.2 ± 15.1, post hoc *p* = 0.027; Figure 5) and the VAS dryness score (55.3 ± 27.2 vs. 28.0 ± 23.9, post hoc *p* = 0.023; Figure 6). In contrast, no significant changes in OSDI or VAS dryness scores were observed in the BRUDER group over time (post hoc *p* ≥ 0.06). There were no significant differences for any of the subjective vision-related items between the two treatment groups (*p* ≥ 0.12) or between visits (*p* ≥ 0.09).

### 3.5. Lipid Layer Thickness 

No significant differences were observed in average, minimum or maximum LLT between the two treatment groups (*p* ≥ 0.24) or between visits (*p* ≥ 0.07).

### 3.6. Adverse Events

Eight mild-to-moderate treatment-related adverse events were reported in the Meiboleyes^®^ group (blurry vision following treatment *n* = 4; burning sensation at the application site during treatment *n* = 3; eyelid redness following treatment *n* = 1), compared to seven in the BRUDER group (blurry vision following treatment *n* = 4 [including the participant who discontinued from the study]; transient burning sensation at the application site during treatment *n* = 1; eyelid redness following treatment *n* = 1; discomfort on lower orbital rim following treatment *n* = 1). All adverse events resolved without sequelae. 

### 3.7. Compliance to Treatment

Self-reported compliance to treatment was calculated as a percentage based on participants’ responses recorded in their daily diary (number of times treatment used ÷ total number of times treatment should have been used × 100). Compliance to the Meiboleyes^®^ treatment was significantly higher compared to the BRUDER group (91.3 ± 8.9 vs. 79.0 ± 11.0%, *p* = 0.015).

## 4. Discussion

This prospective, randomised, parallel group, pilot study demonstrated that the prototype electronic heating device, Meiboleyes^®^, is safe and effective for the treatment of Meibomian gland dysfunction when used twice daily for a duration of six weeks. Expected minor adverse events were consistent with previous reports on eyelid warming devices [10,33], with transient blurry vision most commonly reported (*n* = 4 in each group).

Improvements in meibomian gland function were comparable to the BRUDER Moist Heat Eye Compress. Significant improvements in MGS score have previously been reported after one month of twice-daily use of the BRUDER compress in a contact-lens-wearing population [34]. In contrast, no significant changes were found in MGS or MGYLS following twice-daily use of an alternate microwave-heated eye mask (MGDRx EyeBag) up to eight weeks following treatment [35]. It is possible that this microwave-heated eye mask performed differently due to differences in heat retention or temperature. Indeed, ex vivo experiments have reported that the BRUDER Moist Heat Eye Compress is able to reach higher temperatures and maintain temperatures above 50 °C for the first 6 min, versus the MGDRx EyeBag, where the peak temperature reached was between 40 to 45 °C [24]. Interestingly, the magnitude of improvements in MGS scores (numerical change in average MGS scores between visits) in the lower eyelid observed with Meiboleyes^®^ (9.5 ± 2.8 vs. 16.7 ± 4.1, *p* = 0.002, Baseline vs. 2 weeks) and the BRUDER compress (8.9 ± 2.2 vs. 16.7 ± 5.2, *p* = 0.001, Baseline vs. 2 weeks) in the current study were greater than that reported for three sessions of Intense Pulsed Light therapy combined with meibomian gland expression (IPL+MGX) at four-week intervals (2.3 ± 3.2 vs. 8.2 ± 6.2, *p* < 0.001, Baseline vs. 28 days after the third session) [36]. This difference may be attributed to the timing of outcome measurement - on the same day following the final treatment in the current study versus 28 days after the third session of IPL+MGX. 

Despite the observed improvements in meibomian gland function for both groups, no corresponding changes were detected in lipid layer thickness, meibography, or tear film stability, except for an improvement in fluorescein TBUT at week 6 in the Meiboleyes^®^ group only. Similarly, while significant improvements in MGS scores were observed after four treatments of IPL+MGX followed by multi-frequency radiofrequency, NIKBUT did not change [19]. This may indicate a delayed benefit or downstream effect in tear film integrity, potentially resulting from progressive gland function recovery. While treatment enhanced meibum secretion, improvements in tear film stability and lipid layer composition may require additional time [37], or other factors, such as enhanced meibum distribution or changes in tear dynamics, to manifest. A significant increase in LLT was previously reported immediately following a five-minute treatment with both the BRUDER Moist Heat Eye Compress and a warm towel compress, but the effect was not sustained beyond 15 min post-treatment [38]. These findings indicate that heat-based therapies only temporarily enhance the lipid layer. Other factors, such as meibum composition, play an important role in tear film stability [39] and would be worthwhile to investigate in future studies. 

Symptomatic improvements in OSDI score and VAS subjective dryness scores were observed in the Meiboleyes^®^ group only after 6 weeks of use. No significant changes were observed for the BRUDER compress group in the present study, which contrasts with previous studies on other microwaveable heated masks, where subjective improvement was reported following 2 to 8 weeks of use [34,35,40]. The lower self-reported compliance rate in the BRUDER group, along with variability in microwave power settings, may have influenced efficacy of the BRUDER Compress. The requirement for the BRUDER Moist Heat Eye Compress to be reheated after 7.5 min may have also adversely impacted compliance to treatment. 

The improvements in tear film stability and subjective comfort observed with the Meiboleyes^®^ device may be attributed to the benefits of delivering a constant, sustained therapeutic temperature over the treatment period. Future potential technological advances for the Meiboleyes^®^ device that might assist eye care practitioners with MGD management include the development of an app which could allow monitoring regarding user compliance and temperature monitoring, and implementation of a reminder system to improve compliance. This also offers the potential advantage to eye care practitioners of titrating and customising treatment plans for patients, depending on their individual responses. 

Limitations of the study include the inability to mask participants and lack of control over treatment temperature for the BRUDER control group in this pilot study. It is also uncertain whether participants’ reported compliance to use of the BRUDER compress included reheating the device after 7.5 min, to achieve a total treatment time of 15 min with each use. A potential criticism of the study is the exclusion criteria for LipiFlow treatment within the last six months, given that reports indicate that some effects of LipiFlow can last up to one year [41,42]. However, it was confirmed that none of the participants in the current study received LipiFlow treatment within 12 months prior to participation. In addition, while the recommended treatment for MGD following eyelid warming includes manual massage to facilitate meibum expression to help unblock the glands [6], the subsequent massage step was deliberately omitted in this parallel group study. Given the lack of standardisation as to the appropriate type and length of eyelid massage following mask use [6], this would have confounded the study findings. Further, the effect size for differences in OSDI scores was 1.04, which yielded >95% power for assessing between visit differences and 60% for assessing between group differences. Therefore, this pilot study was underpowered to detect differences between the two groups. Future studies with larger sample sizes would be required to distinguish between the performance of these treatments.

## 5. Conclusions

This pilot study demonstrated that Meiboleyes^®^ is safe and effective for the treatment of meibomian gland dysfunction when used twice daily for a duration of 6 weeks. Improvements in meibomian gland function were comparable to the BRUDER Moist Heat Eye Compress, but significant improvements in subjective comfort after 6 weeks of treatment were observed with the Meiboleyes^®^ device only.

## 6. Patents

nthalmic Pty Ltd. has proprietary interests in commercialising the Meiboleyes^®^ device, which is covered by patent applications (PCT/AU2021/050466, PCT/AU2025/050286), design rights and trademarks across several jurisdictions.

## Figures and Tables

**Table 1:** Description of study treatments.

Study Product	Manufacturer	Composition
Meiboleyes^®^	nthalmic Pty Ltd.	Electronic device containing heating eye cups
BRUDER Moist Heat Eye Compress	Bruder Healthcare Company LLC	Fabric eye mask containing MediBeads

**Table 2:** Baseline characteristics for participants who completed the study.

Baseline Characteristics	Meiboleyes^®^ (*n* = 10)	BRUDER (*n* = 10)	*p*-Values
Gender (Male: Female)	7:3	3:7	−
Age (years)	38.7 ± 14.5	38.9 ± 14.8	0.853
Age range—min to max (years)	28−74	28−75	−
Ethnicity (Non-Asian: Asian)	1:9	1:9	−
Ocular Surface Disease Index score (mean ± SD)	45.2 ± 15.1	38.3 ± 16.4	0.332 *
Visual Analogue Scale-Dryness (0–100)	28.0 ± 23.9	32.3 ± 2.08	0.529
Average lipid layer thickness (nm)	56.8 ± 15.0	65.4 ± 28.0	0.496
Average non-invasive keratography break-up time (s)	9.3 ± 4.8	10.1 ± 5.8	1.000
Tear break-up time (s)	5.5 ± 1.8	7.2 ± 1.7	0.052
Meibomian gland secretion score (0–45)	9.5 ± 2.8	8.9 ± 2.2	0.631
Number of meibomian glands yielding liquid secretion (0–15)	4.5 ± 1.2	3.5 ± 1.3	0.105

* Parametric test.

## Data Availability

The data that support these findings may be made available upon reasonable request.

## Abbreviations

The following abbreviations are used in this manuscript:

MGD	Meibomian Gland Dysfunction
OSDI	Ocular Surface Disease Index
TBUT	Tear Break-up Time
VAS	Visual Analogue Scale
MGS	Meibomian Gland Secretion
MGYLS	Meibomian Glands Yielding Liquid Secretion
IPL	Intense Pulsed Light
MGX	Meibomian Gland Expression
NIKBUT	Non-Invasive Keratograph Break-Up Time
